# Evaluation of the Accuracy of Intraoperative Femoral Stem Anteversion Using a Mechanical Alignment Guide and a CT-Based Navigation System

**DOI:** 10.7759/cureus.100081

**Published:** 2025-12-25

**Authors:** Yoshinobu Masumoto, Shigeo Fukunishi, Takuya Nakai, Toshiya Tachibana

**Affiliations:** 1 Orthopaedic Surgery, Kawasaki Hospital, Kobe, JPN; 2 Orthopaedic Surgery, Nishinomiya Kaisei Hospital, Nishinomiya, JPN; 3 Orthopaedic Surgery, Hyogo Medical University, Nishinomiya, JPN

**Keywords:** ct-based navigation system, femoral stem anteversion, mechanical alignment guide, the absolute discrepancy, total hip arthroplasty

## Abstract

Background

In total hip arthroplasty (THA), implant position is one of the critical factors affecting implant longevity, clinical outcomes, and postoperative complications. Although a number of studies have reported the accuracy of acetabular component positioning using a computed tomography (CT)-based navigation system, the significance of femoral stem anteversion has not been well addressed in the literature.

Methods

Stem anteversion in 83 patients who underwent primary THA and bipolar hemiarthroplasty was simultaneously measured with both a CT-based navigation system and a mechanical alignment guide during surgery. Both intraoperative measurement values were compared with the postoperative measurement values using the same three-dimensional coordinate system.

Results

The absolute discrepancy between the mechanical alignment guide value and the postoperative stem anteversion was 7.6° ± 5.2° (range: 0° to 17°). On the other hand, the absolute discrepancy between the intraoperative stem anteversion value measured with CT-based navigation and the postoperative stem anteversion was 2.9° ± 3.1° (range: 0° to 14°). The absolute discrepancy was significantly lower in intraoperative CT-based navigation (p = 0.001).

Conclusion

Intraoperative stem anteversion measurements using CT-based navigation were more accurate than those using the mechanical alignment guide.

## Introduction

In total hip arthroplasty (THA), implant position is one of the critical factors affecting implant longevity, clinical outcomes, and postoperative complications [1-5]. Although a number of studies have reported an optimal position for the acetabular component and the accuracy of acetabular component positioning using a computed tomography (CT)-based navigation system has also been investigated [6-11], the significance of femoral stem anteversion has not been well addressed in the literature. However, stem anteversion accuracy is just as important a factor in THA longevity as cup alignment [12,13]. In general, the surgeon visually determines the intraoperative stem anteversion angle. However, this conventional procedure can be associated with substantial errors [14]. Additionally, Dorr et al. [15] reported a poor correlation between the surgeons’ visual assessment of stem anteversion and postoperative CT measurements, with an accuracy of 11.3°. Although the accuracy and reproducibility of stem and cup alignment are both expected clinical outcomes, there are very few publications available on the accuracy of stem anteversion in THA using CT-based navigation [16,17]. Hirata et al. [18] and Lee et al. [19] measured intraoperative stem anteversion using a manual goniometer and compared it to postoperative stem anteversion. Discrepancies of less than 5° were reported as 61% and 71%, respectively.

Since 2010, we have developed and utilized the mechanical alignment guide as a simple manual instrument for intraoperative assessment and adjustment of stem anteversion. In our previous study, the effectiveness of this system was examined, comparing it to the postoperative CT evaluation, and it showed that the absolute discrepancy between intra- and postoperative measurements averaged 4.6° ± 4.1° [20]. As a result, it was concluded that the mechanical alignment guide provides reasonable accuracy. Thus, continuing on from our previous findings, the next question would be whether or not the mechanical alignment guide could offer equally accurate measurements in comparison to the CT-based navigation that is currently used in our practice. However, to our knowledge, there are no papers comparing the results and accuracy of stem anteversion between the use of an intraoperative alignment guide and CT-based navigation. The purpose of this study was to compare the accuracy of stem anteversion using the mechanical alignment guide and CT-based navigation in the same postoperative three-dimensional (3D) coordinate system.

## Materials and methods

Study design and population

This research has been approved by the IRB of the authors’ affiliated institutions. This study was a prospective study that was conducted over a limited period of time. The criteria for inclusion in this study were patients who underwent THA and bipolar hemiarthroplasty (BHA) with posterior approach using the same cementless stem (Accolade II, Stryker Orthopedics, NJ, USA). A total of 90 patients who underwent primary THA and BHA between January 2020 and July 2021 were included in this study. We defined the exclusion criteria as patients with intraoperative loosening of the navigation pin, unexplained measurement errors, and a history of total knee arthroplasty (TKA) and unicompartmental knee arthroplasty (UKA) in the ipsilateral lower limbs.

Preoperative planning

All participants underwent a preoperative CT examination from the pelvis to the posterior femoral condyle. At our institution, we generally set the target value of combined anteversion (CA) at 40°-60° for THA with posterior approach, referring to the previous papers by Nakashima et al. [21]. The details of the preoperative planning are as follows: first, using a CT-based navigation system (CT-based hip navigation Version 1.3, Stryker Leibinger, Freiburg, Germany), the cup anteversion was set at around 20°, considering the shape of the acetabulum to avoid anterior protrusion. Next, the target value of stem anteversion is determined in each case by subtracting the cup anteversion angle from the target CA. Preoperative planning was performed using the 3D coordinate system in the software built into the CT-based navigation.

We used a functional pelvic plane (FPP) as the pelvic coordinate system and the table top plane as the femoral coordinate system. As described by Kingsley and Olmsted [22], the table top plane was based on the plane consisting of the posterior margin of the greater trochanter and the bilateral posterior condyles of the femur, serving as the reference plane of the 3D coordinate system, and the z-axis is the projected axis of the posterior condylar plane of a line connecting the trochanteric fossa and the most distal end of the intercondylar region. The femoral neck axis was defined as the transverse slice on the most proximal portion of the inferior neck, without the head portion, as proposed by Sugano et al. [23]. The native femoral anteversion was defined as the angle between the femoral neck axis and the tabletop plane.

Surgical procedures and measurement

One surgeon performed the THA and BHA procedures using a conventional posterior approach with the patients in the lateral decubitus position in all cases. CT-based navigation was used to determine both the cup and stem alignment. All hips were implanted with a cementless stem. During surgery, the femoral anteversion value was determined at the final rasping of the femur, and the mechanical alignment guide and CT-based navigation were used to simultaneously measure the stem anteversion angle.

**Figure 1:**
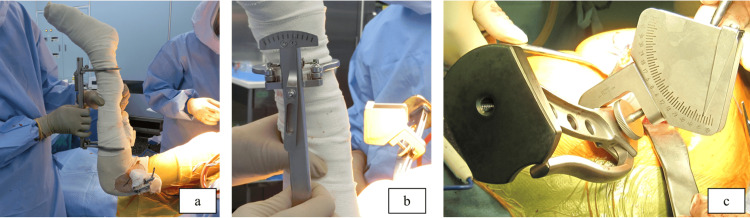
Mechanical alignment guide consists of two parts Photograph showing the intraoperative setup of the mechanical alignment guide. The external rotation of the femur at 90° of knee flexion was taken into consideration (a, b). Stem anteversion was measured (c).

The structure and concept of the mechanical alignment guide are described as follows. The part of the mechanical alignment guide attached to the lower leg is used to ascertain the perpendicularity of the lower leg axis. The other part provides information for the orientation of the final rasp. The intraoperative setup is shown in Figure 1.

The lower leg is held perpendicular to the floor at 90° hip flexion and 90° internal rotation. At this time, confirm that the mechanical alignment guide attached to the lower leg is perpendicular to the floor (indicating 0°). The mechanical alignment guide value attached to the rasp handle can be used to intraoperatively measure the stem anteversion angle.

The correction angle, also called the condylar twist angle, was defined as the angle between the clinical epicondylar axis (CEA) and the posterior condyle line in preoperative CT [24]. The intraoperative stem anteversion measured by the alignment guide was defined as the sum of the intraoperative measured value and the correction angle, which was defined as “the corrected alignment guide value.”

**Figure 2:**
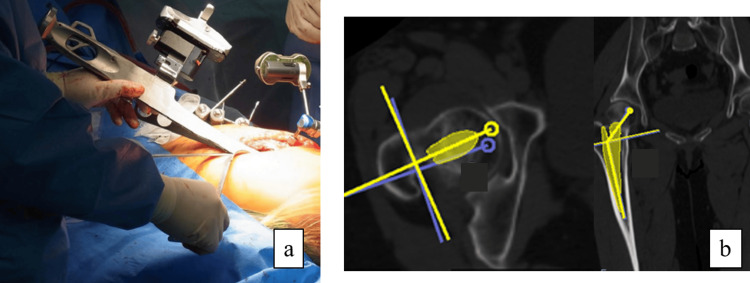
Stem anteversion angle measured using CT-based navigation Intraoperative setup of the CT-based navigation system (a). Postoperative stem anteversion measured using the CT-based navigation workstation (b). CT, computed tomography

Intraoperative measurement of stem anteversion using CT navigation was performed during final rasping by measuring the angle formed between the tracker device attached to the pin inserted into the femoral condyle and the tracker device attached to the reamer handle, with the FPP serving as the reference plane (Figure 2).

Postoperative evaluations

All patients included in this study underwent post-operative CT examinations approximately one week after surgery. Postoperative stem anteversion was measured by superimposing a virtual illustration of the stem onto the actual component of the postoperative CT images at the CT-based navigation workstation (Figure 2).

In addition, postoperative stem anteversion was defined as the angle formed between the femoral stem axis and the tangential line to the bilateral posterior femoral condylar margin on the tabletop plane of the CT-based navigation workstation. Stem sagittal alignment and coronal alignment were also measured using the same coordinate system. All CT-based navigation measurements of postoperative stem anteversion were performed by the same observer. Measurements were repeated in a blind manner over the course of two sessions, which were at least two months apart. The primary outcome was assessed by comparing the values of stem anteversion using CT-based navigation and the mechanical alignment guide during surgery with postoperative measurement values, which were defined as the true stem anteversion. The secondary outcome evaluated factors that affected the accuracy of the intraoperative stem anteversion values measured by the mechanical alignment guide, as compared to true stem anteversion values.

Statistical analysis

Power was calculated a priori using the absolute discrepancy between the two intraoperative measured values and the postoperative measured value of stem anteversion in the first 20 cases. A sample of 78 participants provided the effect size 0.83, statistical power 0.95, and alpha error 0.05. All statistical analyses were conducted using IBM SPSS Statistics for Windows, Version 26 (Released 2018; IBM Corp., Armonk, NY, USA). Continuous data were analyzed using the nonparametric Student’s t-test, with p < 0.05 considered significant. Multivariate linear regression analyses were performed to analyze the potential risk factors that might affect the discrepancy between the two measurements.

## Results

There were 90 patients (90 hips) who enrolled in the study in which THA and BHA were performed. Seven patients were excluded from the study as a result of the exclusion criteria. A total of seven cases were excluded from this study according to each of the following criteria: one hip with intraoperative loosening of the navigation pin, one hip with unexplained measurement errors, and five hips with a history of TKA or UKA in the ipsilateral lower limbs. Therefore, a total of 83 primary THA in 43 (51.8%) hips and BHA in 40 (48.2%) hips were included in the final analysis. Out of those 83 hips, there were 23 (27.7%) male and 60 (72.3%) female patients, with a mean age of 81.5 ± 8.4 years (range: 52 to 91 years). Pre-operative diagnosis included primary osteoarthritis (OA) in two (2.4%) hips, developmental dysplasia in 35 (42.2%) hips, osteonecrosis of the femoral head in three (3.6%) hips, and femoral neck fracture in 43 (51.8%) hips. Table 1 shows the patient demographics and diagnostic data.

**Table 1:** Patient characteristic data * indicates mean ± SD (range).

Parameters	Results
Age (year)	81.5 ± 8.4 (52-91)*
Gender (male/female)	23/60
Height (m)	1.55 ± 0.95 (1.37-1.80)*
Weight (kg)	48.0 ± 9.9 (32.5-83.1)*
Body mas index (kg/m²)	19.9 ± 3.1 (11.8-29.4)*
Bone mineral density (g/cm²)	
Lumber vertebra	0.890 ± 0.189 (0.557-1.446)*
Proximal femur	0.644 ± 0.108 (0.382-0.891)*
The Kellgren and Lawrence classification	
Grade 1	14 (16.9%)
Grade 2	51 (61.4%)
Grade 3	14 (16.9%)
Grade 4	4 (4.8%)

Native femoral anteversion and preoperative target stem anteversion in the navigation system were averaged at 18.1° ± 8.5° (range: 0°-36°) and 23.4° ± 8.1° (range: 8°-40°), respectively. The condylar twist angle, composed between the CEA and the posterior condyle line, was an average of 5.2° ± 1.4° (range: 2°-8°) (Table 2).

**Table 2:** Results of stem anteversion All values are expressed as mean ± SD (range). CT, computed tomography

Parameters	Angle (range)
Preoperative planning	
Native femoral anteversion	18.1° ± 8.5° (0°-36°)
Preoperative target stem anteversion	23.4° ± 8.1° (8°-40°)
Condylar twist angle	5.2° ± 1.4° (2°-8°)
Intraoperative measurement	
Mechanical alignment guide	27.5° ± 10.0° (2°-46°)
Corrected alignment guide value	32.7° ± 10.4° (8°-52°)
CT-based navigation	27.1° ± 10.4° (4°-56°)
Postoperative measurement	
True stem anteversion	25.0° ± 10.4° (4°-54°)
Conventional measurement method	28.8° ± 11.0° (3°-58°)

**Figure 3:**
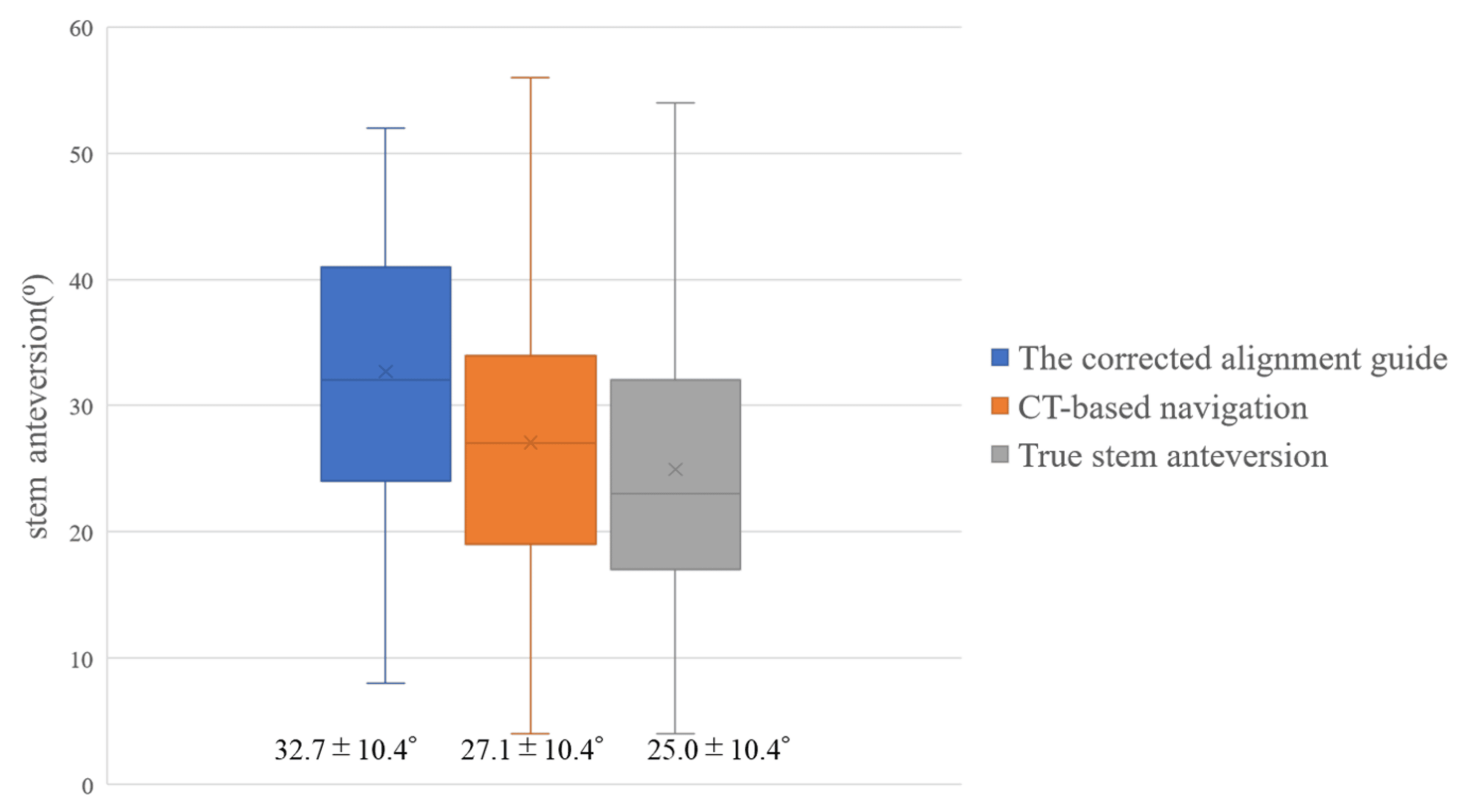
Boxes show intraoperative stem anteversion of the corrected alignment guide and CT-based navigation, and postoperative true stem anteversion The line in the center of the boxes represents the median value, and the bar represents the range of stem anteversion. CT, computed tomography

The average stem anteversion measured by the intraoperative mechanical alignment guide was 27.5° ± 10.0° (range: 2° to 46°), and the corrected alignment guide value was 32.7° ± 10.4° (range: 8° to 52°). The average stem anteversion measured by the intraoperative CT-based navigation was 27.1° ± 10.4° (range: 4° to 56°). Postoperative true stem anteversion was 25.0° ± 10.4° (range: 4° to 54°) (Figure 3).

Postoperative stem anteversion measured using the conventional measurement method in the postoperative CT image was 28.8° ± 11.0° (range: 3° to 58°) (Table 2). The average stem sagittal alignment and coronal alignment were 7.9° ± 2.3° (range: 3° to 11°) varus tilt and 0.6° ± 1.7° (range: -3° to 4°) anterior tilt, respectively.

Intra- and inter-observer reliability were evaluated using the intraclass correlation coefficient (ICC). The ICC for intra- and inter-observer reliability of the postoperative anteversion measurements were 0.992 and 0.986, respectively.

Primary outcome

The absolute discrepancy between the corrected alignment guide value and the true stem anteversion was 7.6° ± 3.8° (range: 0° to 17°). On the other hand, the absolute discrepancy between the intraoperative stem anteversion value measured with CT-based navigation and the true stem anteversion was 2.9° ± 3.1° (range: 0° to 14°). The absolute discrepancy was significantly lower with intraoperative CT-based navigation (p = 0.001) (Table 3).

**Table 3:** Results of stem anteversion and the absolute discrepancy *p < 0.05 considered statistically significant. All values are expressed as mean ± SD. CT, computed tomography

Parameters	Corrected alignment guide	CT-based navigation	True stem anteversion	p-value
Stem anteversion	32.7° ± 10.4°	27.1° ± 10.4°	25.0° ± 10.4°	-
Absolute discrepancy	7.6° ± 3.8°	2.9° ± 3.1°	-	0.001*

**Figure 4:**
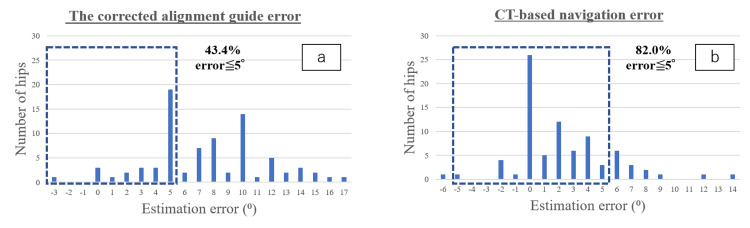
Distribution of errors in intraoperative estimation from postoperative true stem anteversion (a) The corrected alignment guide error was 43.4%, and (b) the CT-based navigation error was 82.0%. CT, computed tomography

Additionally, the measurement accuracy was within 5° in 36 hips (43.4%) and within 10° in 68 hips (82.0%) for the corrected alignment guide value. The measurement accuracy was within 5° in 67 hips (80.7%) and within 10° in 80 hips (96.4%) using CT-based navigation (Figure 4).

**Figure 5:**
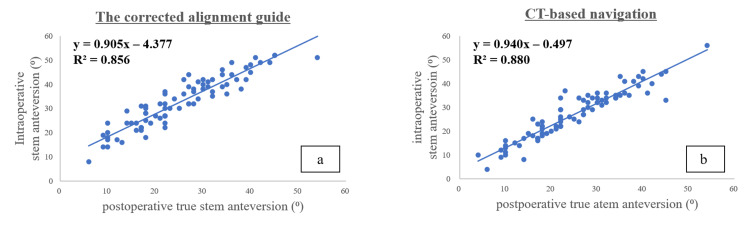
Correlation between intraoperative and postoperative stem anteversion measurements A solid line shows the relationship between intraoperative stem anteversion and postoperative true stem anteversion: (a) r = 0.925, (b) r = 0.938. CT, computed tomography

The Pearson correlation coefficient, which shows the relationship between true stem anteversion and intraoperative stem anteversion, was 0.925 for the corrected alignment guide value and 0.938 for CT-based navigation, indicating a high correlation between the two (Figure 5).

Secondary outcome

Multiple regression analyses revealed that patient weight was significantly related to measurement accuracy, with p = 0.027 (Table 4).

**Table 4:** Multivariate linear regression analysis to identify factors affecting the discrepancy between intraoperative corrected alignment guide measurements and postoperative true stem anteversion *p < 0.05 considered statistically significant; B denotes unstandardized coefficient; SE denotes standard error; β denotes standardized coefficient. BMD, bone mineral density

Parameters	B	SE	β	t	p-value
Age	0.059	0.072	0.129	0.822	0.414
Gender	-1.064	1.308	-0.125	-0.814	0.419
Height	-0.413	0.252	-1.021	-1.639	0.106
Weight	0.899	0.398	2.335	2.259	0.027*
Body mass index	-1.826	0.969	-1.461	-1884	0.064
The Kellgren and Lawrence classification	0.337	1.017	0.037	0.331	0.741
BMD in the lumbar vertebra	-3.835	3.443	-0.185	-1.114	0.269
BMD in the proximal femur	-8.158	5.670	-0.218	-1.439	0.155

## Discussion

In recent years, THA has used CA, which is the sum of the cup and stem anteversion, as a parameter to assess the adequacy of overall prosthetic alignment [12,13,21,25,26]. In 2010, to achieve consistency in stem anteversion, we developed the original mechanical alignment guide for intraoperative assessment and adjustment. Similarly, Pongkunakorn et al. [27] also developed an alignment guide with a similar concept. The addition of a spirit level to check the verticality of the lower leg axis was reported to significantly improve measurement accuracy. In the present study, the intraoperative stem anteversion was simultaneously determined by CT-based navigation and measured by the mechanical alignment guide. For the postoperative measurement of stem anteversion, a computer-aided design model of the stem was superimposed onto the actual implant in postoperative CT images at the same CT-based navigation workstation, and stem anteversion was defined as the angle formed between the proximal femoral stem axis and the tabletop plane. Regarding CT-based navigation, the absolute discrepancy between the intraoperative CT-based navigation value and the true stem anteversion was 2.9° ± 3.1°, with an acceptable accuracy of >5° and >10° in 80.7% and 96.4% of the cases, respectively. The results of this study were as satisfactory as previous reports on the accuracy and usefulness of intraoperative assessment and adjustment of stem anteversion using CT-based navigation [16,28,29]. Regarding the mechanical alignment guide, although the correlation between the corrected alignment guide value and the true stem anteversion showed a high correlation coefficient of 0.925, the absolute discrepancy between the corrected alignment guide value and the true stem anteversion was 7.6° ± 3.8°. The absolute discrepancy was significantly lower with the intraoperative CT-based navigation value. Additionally, the measurement accuracy was within 5° in 43.4% of cases, indicating that it is difficult to achieve accuracy within 5°, even with the use of the mechanical alignment guide.

Several papers have previously reported on the factors affecting the measurement error of intraoperative alignment guides using multivariate regression analysis. Mitsutake et al. [30] and Hirata et al. [18] proposed that the measurement error was significantly influenced by the severity of knee OA. Likewise, Pongkunakorn et al. [27] proposed that the tibiofemoral angle (varus knee deformity) was also a significant influential factor. However, this study found no significant relationship between the severity of knee OA and measurement error. Lee et al. [19] reported that only the tibiofemoral angle significantly affected the measurement error and found no association with the severity of knee OA. Knee OA severity was evaluated using the Kellgren and Lawrence (KL) classification. Since the KL classification of knee OA does not assess the degree of varus deformity, it is possible that the subjects in this study included some cases of knee OA without varus deformity, and that the severity of knee OA may not have contributed to measurement accuracy. On the other hand, patient body weight was found to be significantly related to measurement accuracy (p = 0.027). In cases of a posterior approach for heavier patients, it may have been difficult for the surgical assistant to hold the limb position, as described above, with the guide attached to the lower leg when measuring stem anteversion. The risk factors for measurement errors using the mechanical alignment guide should also be considered along with other approaches.

There was a limitation present in this study. First, intraoperative measurement using the mechanical alignment guide did not include stem sagittal alignment. Hirata et al. [18] examined the discrepancy between stem anteversion and native femoral anteversion and found that anteriorly tilting the sagittal stem alignment by 1° reduced the discrepancy (stem anteversion - native femoral anteversion) by 2.3°. The anterior tilt of the stem sagittal alignment could be one of the contributing factors to the measurement error of the mechanical alignment guide. In this study, the mechanical alignment guide showed an absolute discrepancy of 7.6° ± 3.8° compared to the postoperative stem anteversion angle, which was inferior to CT navigation. However, in actual clinical practice, we do not completely reject the use of mechanical alignment guides. In facilities without navigation systems, we consider mechanical alignment guides useful as a simple method to determine stem anteversion intraoperatively. In the future, we believe that detailed studies are needed on the relationship between stem sagittal alignment and stem anteversion, as well as on standardizing the position of the lower limbs during surgery.

Second, this study examined surgeries performed by a single surgeon at a single institution, which may have resulted in bias and problems with reproducibility.

Finally, in this case series, there were no events attributable to implant positioning during the observation period, such as dislocations or cases reporting groin pain thought to be due to iliopsoas impingement. However, this study did not evaluate the impact of stem anteversion accuracy on clinical outcomes. Future studies are needed to investigate the relationship between the accuracy of stem anteversion using CT navigation and postoperative clinical outcomes.

## Conclusions

The present study revealed that intraoperative measurement of stem anteversion with both CT-based navigation and the mechanical alignment guide showed an excellent correlation with the true stem anteversion. Measurement of intraoperative stem anteversion using CT-based navigation was more accurate than the method using the mechanical alignment guide.

## References

[1] Lewinnek GE, Lewis JL, Tarr R, Compere CL, Zimmerman JR (1978). Dislocations after total hip-replacement arthroplasties. J Bone Joint Surg Am.

[2] Widmer KH, Zurfluh B (2004). Compliant positioning of total hip components for optimal range of motion. J Orthop Res.

[3] Yoshimine F (2006). The safe-zones for combined cup and neck anteversions that fulfill the essential range of motion and their optimum combination in total hip replacements. J Biomech.

[4] Sugano N, Nishii T, Miki H, Yoshikawa H, Sato Y, Tamura S (2007). Mid-term results of cementless total hip replacement using a ceramic-on-ceramic bearing with and without computer navigation. J Bone Joint Surg Br.

[5] Miki H, Yamanashi W, Nishii T, Sato Y, Yoshikawa H, Sugano N (2007). Anatomic hip range of motion after implantation during total hip arthroplasty as measured by a navigation system. J Arthroplasty.

[6] Kajino Y, Kabata T, Maeda T, Iwai S, Kuroda K, Tsuchiya H (2012). Does degree of the pelvic deformity affect the accuracy of computed tomography-based hip navigation?. J Arthroplasty.

[7] Iwana D, Nakamura N, Miki H, Kitada M, Hananouchi T, Sugano N (2013). Accuracy of angle and position of the cup using computed tomography-based navigation systems in total hip arthroplasty. Comput Aided Surg.

[8] Kuroda K, Kabata T, Maeda T (2014). The value of computed tomography based navigation in revision total hip arthroplasty. Int Orthop.

[9] Maeda Y, Sugano N, Nakamura N, Hamawaki M (2015). The accuracy of a mechanical cup alignment guide in total hip arthroplasty (THA) through direct anterior and posterior approaches measured with CT-based navigation. J Arthroplasty.

[10] Tetsunaga T, Yamada K, Tetsunaga T, Sanki T, Kawamura Y, Ozaki T (2020). An accelerometer-based navigation system provides acetabular cup orientation accuracy comparable to that of computed tomography-based navigation during total hip arthroplasty in the supine position. J Orthop Surg Res.

[11] Nishihara S, Hayashida K (2022). Comparison between freehand technique and computed tomography-based navigation in acetabular cup placement through direct anterior approach for total hip arthroplasty. Arch Orthop Trauma Surg.

[12] Masumoto Y, Fukunishi S, Fukui T (2020). New combined anteversion technique in hybrid THA: cup-first procedure with CT-based navigation. Eur J Orthop Surg Traumatol.

[13] Fukunishi S, Nishio S, Fujihara Y, Okahisa S, Takeda Y, Fukui T, Yoshiya S (2016). Accuracy of combined anteversion in image-free navigated total hip arthroplasty: stem-first or cup-first technique?. Int Orthop.

[14] Wines AP, McNicol D (2006). Computed tomography measurement of the accuracy of component version in total hip arthroplasty. J Arthroplasty.

[15] Dorr LD, Wan Z, Malik A, Zhu J, Dastane M, Deshmane P (2009). A comparison of surgeon estimation and computed tomographic measurement of femoral component anteversion in cementless total hip arthroplasty. J Bone Joint Surg Am.

[16] Kitada M, Nakamura N, Iwana D, Kakimoto A, Nishii T, Sugano N (2011). Evaluation of the accuracy of computed tomography-based navigation for femoral stem orientation and leg length discrepancy. J Arthroplasty.

[17] Hayashi S, Nishiyama T, Fujishiro T (2013). Evaluation of the accuracy of femoral component orientation by the CT-based fluoro-matched navigation system. Int Orthop.

[18] Hirata M, Nakashima Y, Ohishi M, Hamai S, Hara D, Iwamoto Y (2013). Surgeon error in performing intraoperative estimation of stem anteversion in cementless total hip arthroplasty. J Arthroplasty.

[19] Lee YK, Kim JW, Kim TY, Ha YC, Koo KH (2018). Validity of the intra-operative measurement of stem anteversion and factors for the erroneous estimation in cementless total hip arthroplasty using postero-lateral approach. Orthop Traumatol Surg Res.

[20] Fujihara Y, Fukunishi S, Fukui T (2016). Use of the G-guide for measuring stem antetorsion during total hip arthroplasty. Orthopedics.

[21] Nakashima Y, Hirata M, Akiyama M (2014). Combined anteversion technique reduced the dislocation in cementless total hip arthroplasty. Int Orthop.

[22] Kingsley PC, Olmsted KL (1948). A study to determine the angle of anteversion of the neck of the femur. J Bone Joint Surg Am.

[23] Sugano N, Noble PC, Kamaric E (1998). A comparison of alternative methods of measuring femoral anteversion. J Comput Assist Tomogr.

[24] Yoshioka Y, Siu D, Cooke TD (1987). The anatomy and functional axes of the femur. J Bone Joint Surg Am.

[25] Amuwa C, Dorr LD (2008). The combined anteversion technique for acetabular component anteversion. J Arthroplasty.

[26] Dorr LD, Malik A, Dastane M, Wan Z (2009). Combined anteversion technique for total hip arthroplasty. Clin Orthop Relat Res.

[27] Pongkunakorn A, Phetpangnga N, Kananai N (2021). Accuracy of intraoperative estimation of femoral stem anteversion in cementless total hip arthroplasty by using a digital protractor and a spirit level. J Orthop Surg Res.

[28] Nakahara I, Kyo T, Kuroda Y, Miki H (2018). Effect of improved navigation performance on the accuracy of implant placement in total hip arthroplasty with a CT-based navigation system. J Artif Organs.

[29] Nakahara I, Kyo T, Kuroda Y, Miki H (2021). Does difference in stem design affect accuracy of stem alignment in total hip arthroplasty with a CT-based navigation system?. J Artif Organs.

[30] Mitsutake R, Tanino H, Nishida Y, Higa M, Ito H (2020). A simple angle-measuring instrument for measuring cemented stem anteversion during total hip arthroplasty. BMC Musculoskelet Disord.

